# Investigating the effects of previous injury on subsequent training loads, physical fitness, and injuries in youth female basketball players

**DOI:** 10.3389/fphys.2025.1506611

**Published:** 2025-01-23

**Authors:** Yuanqi Huang, Shaonan Wang, Changfei Li, Yukun Wang, Zhanshuang Bai, Binghao Lv, Yuheng Gui, Zhongjian Wei

**Affiliations:** ^1^ School of Physical Education and Sport Science, Fujian Normal University, Fuzhou, China; ^2^ Fujian Provincial Basketball and Volleyball Sports Management Center, Fuzhou, China; ^3^ School of Sport and Recreation, Auckland University of Technology, Auckland, New Zealand; ^4^ Faculty of Sport Science and Technology, Bangkok Thonburi University, Bangkok, Thailand; ^5^ School of Tourism and Sports Health, Hezhou University, Hezhou, China; ^6^ School of Teacher Education, Hezhou University, Hezhou, China

**Keywords:** training load, physical fitness, injury, youth, basketball

## Abstract

**Background:**

Previous studies have shown that athletes accustomed to higher chronic workloads are less susceptible to injury than those exposed to lower chronic workloads. However, few studies have evaluated whether previous injury influences them. Therefore, this study investigated the impact of previous injuries on subsequent training loads, physical fitness, and injury rates in female youth basketball players.

**Methods:**

Training load, physical fitness, and injuries of 18 young female basketball players (age 16.8 ± 1.4 years) were monitored. Previous injury status was clustered using the K-means clustering algorithm to separate players into high-risk and low-risk groups. Linear mixed models were used to analyze the effects of previous injury status on subsequent training load and physical fitness. Meanwhile, the differences between the players’ injury groups were analyzed.

**Results:**

Previous injury status can significantly impact a player’s subsequent training loads, including acute loads, chronic loads, skill-based training loads, training monotony, and training strain (all *p* < 0.05). The two groups had no significant differences in physical fitness (all *p* > 0.05). Furthermore, the incidence of non-contact injuries was significantly higher in the high-risk group than low-risk group, which would result in more training time lost (all *p* < 0.05).

**Conclusion:**

This study identified the impact of previous injury status on subsequent training load, physical fitness, and injuries in youth female basketball players. These findings provide valuable insight for coaches to optimize training loads according to previous injury status, aiming to minimize the likelihood of subsequent injuries.

## 1 Introduction

In recent years, there has been a growing emphasis on specialization in elite youth sports, leading to a level of competitiveness akin to adult sports (Bergeron et al., 2024). While this approach may help identify young athletes with the potential to become elite athletes, it is important to recognize that an overemphasis on early professional success may lead to a series of health problems. For example, Hall and colleagues found that female adolescents who engaged in early sports specialization exhibited a markedly higher risk of knee health problems (Hall et al., 2015). Additionally, previous experience has indicated that during periods of rapid growth, long bones lengthen more quickly than the muscle-tendon complex, which can create tension on the tendon apophysis and potentially lead to traction injuries on the growth plate (Nguyen and Caine, 2024). Consequently, these adverse outcomes not only hinder subsequent training and impede physical fitness development but also interfere with the healthy growth of youth athletes (Bahr, 2014).

High training volume, intensity, and competition frequency are critical to improving competitive performance in young athletes, but they also raise concerns about sports-related injuries (Visnes and Bahr, 2013). It is commonly believed that higher training loads lead to more injuries. However, recent research suggests that athletes accustomed to high training loads experience fewer injuries than those with lower training loads, which contradicts conventional wisdom (Hulin et al., 2016). For instance, Gabbett and colleagues found that rapid increases in training load significantly raised the likelihood of injuries in team sports (Gabbett et al., 2016). Then, they introduced the concept of the acute-to-chronic workload ratio (ACWR) to measure these rapid changes in training loads and proposed the training-injury prevention paradox (Gabbett, 2016). They suggest that players who experience high chronic loads have developed sports-matched physical fitness, which reduces the risk of injury associated with a sudden increase in training load. Despite the appeal of this paradox, the conflicting findings of the available investigations make determining the relationship between training load and injury remains an open question (Mohr et al., 2023; Impellizzeri et al., 2020). Consequently, these findings prompt the interesting question: “What factors contribute to individual differences in tolerance for rapid spikes in load? (Gabbett, 2020)”

Previous injury, which is a significant risk factor with strong evidence in elite youth sports, can lead to problems such as reduced muscle strength, altered muscle recruitment patterns and proprioceptive deficits (Fulton et al., 2014). For instance, Røksund and colleagues demonstrated that football players who experienced hamstring strains exhibited a significant decrease in speed during a repetitive sprint test (Røksund et al., 2017). Similarly, Buhmann and his team found that hamstring injury history was associated with long-term deficits in autonomic activation during maximal centrifugal contraction, stretch reflex, and tendon reflex amplitude (Buhmann et al., 2020). This evidence highlights that previous injuries can significantly alter internal risk factors, thereby directly or indirectly impacting an athlete’s training tolerance and physical fitness (Alonso-Muñoz et al., 2023; Steib et al., 2013). Although most existing research on previous injury history has investigated factors that may influence new injuries, such as changes in kinematics and motor skills, few studies have focused on the impact of previous injuries on subsequent training, which may blur the association between training load and injury (Sedeaud et al., 2020). Hence, it is necessary to investigate the impact of previous injuries on subsequent training.

With this in mind, this study utilizes metadata from previously published research to preliminarily investigate the effects of previous injuries on subsequent training load, physical fitness, and injury in young female basketball players (Huang et al., 2022). It was hypothesized that players with a higher injury burden would be characterized by an inability to withstand higher training loads (both accumulation and variability), poorer fitness, and a higher incidence of injuries than players with a lower injury burden.

## 2 Materials and methods

### 2.1 Ethics statement

This study was conducted following the guidelines of the Declaration of Helsinki and was approved by the Fujian Provincial Basketball and Volleyball Sports Management Center (protocol number: FJBVSM2020101001).

### 2.2 Participants

Eighteen youth female basketball players (age: 16.8 ± 1.4 years, height: 175.7 ± 6.0 cm, weight: 66.1 ± 6.0 kg, training years: 3.4 ± 1.7 years) were recruited. Training load, physical fitness, and injury monitoring data were collected from participants over 20 weeks. The team’s weekly schedule consisted of 5–6 skill-based basketball sessions lasting 80–120 min, emphasizing skills development and game-based conditioning. Additionally, the participants engaged in 2–3 physical training sessions lasting 60–80 min per week, focusing on metabolic conditioning, strength/power, and agility development. Before the commencement of the study, all participants were provided with written and oral information about the study and were required to sign an informed consent form.

### 2.3 Training load

The Borg-10 ratings of perceived exertion (RPE) scale was used to monitor the perceived exertion experienced by the players following each training session, which has been confirmed for its validity (Foster et al., 1995; Chen et al., 2002). Then, the duration between the start and end of the training was recorded. The internal training load was quantified by calculating the session rating of perceived exertion (sRPE) using Equation 1.

sRPE=duration×RPE (1)

The unit of measurement for the duration was minutes, while the RPE was expressed in arbitrary units (AU). This study assessed the training load information of the athletes by calculating acute training load (ATL), chronic training load (CTL), acute chronic workload ratio (ACWR), monotony, and strain. ATL is the rolling average of sRPE or duration for the last week, and CTL is the rolling average of sRPE or duration for the past 4 weeks. The ACWR, monotony, and strain were calculated as shown in Equations 2–4.

$$ACWR=\frac{ATL}{CTL}$$ (2)

$$Monotony=\frac{Average\;of\;weekly\;training\;load}{Standard\;deviation\;of\;weekly\;training\;load}$$ (3)

Strain=Average of weekly training load×Monotony (4)

### 2.4 Physical fitness

This study used several field-based tests to assess physical fitness according to the testing protocols available in Sports Injury Management (Joyce and Lewindon, 2016). In detail, the squat one-repetition maximum (1 RM) test was utilized to assess the maximal strength of the lower extremities, while the bench press 1 RM test was employed to evaluate the maximal strength of the upper extremities. Agility was evaluated using the 5.8 m × 6 rounds shuttle run, and repeated sprint performance was measured through the 15 m × 17 rounds shuttle run. The run-up vertical jump (RVJ) test measured the explosive and jumping ability.

### 2.5 Injury surveillance

Injury was defined as an event during training or a match that resulted in the player’s absence from subsequent sessions, and injury data were collected according to a standardized collection procedure (Bahr et al., 2020; Fuller et al., 2006). The injury records included details on the injury’s location, nature, time loss, type, occurrence of injury (contact, non-contact), and diagnosis mode. According to the Strengthening the Reporting of Observational Studies in Epidemiology Sports Injury and Illness Surveillance (STROBE-SIIS), this study defined non-contact injuries as injuries caused by indirect contact mechanisms, including overuse and chronic injuries (Bahr et al., 2020). Among them, the upper extremity included the shoulder, upper arm, elbow, forearm, wrist, and hand; the lower extremity included the hips, thighs, knees, calves, ankles, and feet; the trunk included the chest, thoracic spine, lumbosacral, and abdomen. The injury incidence during the investigation was calculated using the following Equation 5:

$$Incidence=\frac{number\;of\;injuries}{training\;exposure}\times 1000\;h$$ (5)

The injury record in the year before the research started was considered the previous injury. Six indicators were incorporated as analysis variables for the cluster, which encompassed the count of upper extremity injury (UEI), the count of lower extremity injury (LEI), the count of trunk injury (TI), loss of time due to upper extremity injuries (LT-UEIs), loss of time due to lower extremity injuries (LT-LEIs), and loss of time due to trunk injuries (LT-TIs).

### 2.6 Statistical analysis

In this study, the K-means clustering algorithm was used to analyze the previous injury history. Prior to clustering, scaling was performed using the median and interquartile range. The optimal number of clusters (k-value) is determined by silhouette coefficient maximization, where the k-value ranges from 2 to 5. Differences in categorical variables were analyzed using the Chi-squared test, while differences in continuous numerical variables were analyzed using Welch’s t-test or Welch’s analysis of variance (It depends on the optimal number of clusters). Linear mixed models were used to assess the effects of time and previous injury history status on dependent variables, treating time and group as fixed effects and player as a random effect. Since this investigation focused on the impact of previous injuries on each indicator of players, only the main and interaction effect of previous injury status on the dependent variable was reported. Post-hoc analyses with Bonferroni correction were conducted for statistically significant main and interaction effects. All hypothesis tests were two-tailed with a significance level (α) of 0.05, and *p*-values greater than 0.1 were considered insignificant, *p*-values less than 0.1 were marginally significant, and *p*-values less than 0.05 were considered significant. The data were analyzed using Jamovi software (version 1.8.1) and Python 3.6 programming environment.

## 3 Results

### 3.1 Previous injury grouping

The clustering results indicate that the maximum silhouette coefficient (0.398) was achieved when the number of clusters (k) was set to 2. As a result, this study divided the players into two clusters. The descriptive statistics for these two clusters are presented in Table 1. It is clearly seen that the two clusters are significantly different in terms of training years, LEI count, LT-LEIs, UEI count, and LT-UEIs (*p* < 0.05), while there were no significant differences in terms of position, age, height, weight, BMI, TI count, and LT-TIs (*p* > 0.05). Consequently, this study named the clusters as high-risk and low-risk groups.

**TABLE 1:** The descriptions and differences in the basic information between the high-risk and low-risk groups.

	High risk	Low risk	*p*	MD	95% CI of MD	Cohen’s d effect size
Position						
Forward	5 (45.5)	5 (71.4)	0.557	—	—	
Center	3 (27.3)	1 (14.3)				
Guard	3 (27.3)	1 (14.3)				
Age (years)	16.91 ± 1.14	16.57 ± 1.90	0.682	0.338	−1.472 to 2.147	0.216
Height (cm)	177.00 ± 6.40	174.00 ± 5.50	0.421	2.351	−3.711 to 8.413	0.394
Weight (kg)	68.10 ± 5.90	62.90 ± 5.10	0.065	5.234	−0.378 to 10.845	0.951
BMI (kg/m^2^)	21.80 ± 1.80	20.70 ± 1.50	0.156	1.144	−0.489 to 2.777	0.707
Training Years (years)	3.55 ± 0.69	2.29 ± 1.25	0.040*	1.260	0.075 to 2.444	1.246
LT-TIs (days)	1.73 ± 2.57	0.86 ± 2.27	0.464	0.870	−1.607 to 3.347	0.359
LT-LEIs (days)	4.19 ± 1.00	0.29 ± 0.76	< 0.001**	3.905	3.023 to 4.787	4.419
LT-UEIs (days)	2.82 ± 1.54	1.00 ± 1.73	0.043*	1.818	0.066 to 3.570	1.110
TI Count	0.64 ± 0.81	0.29 ± 0.76	0.367	0.351	−0.457 to 1.159	0.448
LEI Count	2.55 ± 1.29	0.14 ± 0.38	< 0.001**	2.403	1.502 to 3.304	2.522
UEI Count	1.36 ± 0.92	0.43 ± 0.79	0.037*	0.935	0.064 to 1.807	1.089

Abbreviations: BMI, body mass index; TI, trunk injury; LEI, lower extremity injury; UEI, upper extremity injury; LT-TIs, loss of time due to trunk injuries; LT-LEIs, loss of time due to lower extremity injuries; LT-UEIs, loss of time due to upper extremity injuries; MD, mean difference.

Note: The Chi-squared test was used to compare the position of the two groups; a Welch’s t-test was used to compare age, height, weight, BMI, training year, TI, count, LT-TIs, LEI, count, LT-LEIs, UEI, count, and LT-UEIs.

Bold indicates statistically significant values (p < 0.05).

*p < 0.05.

**p < 0.01.

### 3.2 Training load

The player’s previous injury status did not significantly affect their weekly fitness-based training load and skill-to-fitness training load ratio (*p* > 0.05). However, it did significantly reduce the player’s ATL (*p* = 0.033, Bonferroni corrected), CTL (*p* = 0.021, Bonferroni corrected), and total weekly skill-based training load (*p* = 0.031, Bonferroni corrected). Similar findings were also observed in the analysis of training duration, which showed that previous injury status would significantly decrease a player’s ATL (*p* = 0.034, Bonferroni corrected), CTL (*p* = 0.018, Bonferroni corrected) and total weekly skill-based training session duration (*p* < 0.001, Bonferroni corrected).

There was no significant interaction between previous injury status and time on training load indicators (*p* > 0.05). However, it is important to note that both training monotony (sRPE-based monotony: *p* = 0.004, Bonferroni corrected, duration-based monotony: *p* = 0.002, Bonferroni corrected) and training strain (sRPE-based strain: *p* = 0.008, Bonferroni corrected, duration-based strain: *p* = 0.001, Bonferroni corrected), which were calculated based on sRPE or duration, were significantly lower in the high-risk group (Table 2).

**TABLE 2:** Linear mixed model results for the effect of previous injury status on training load and physical fitness.

Dependent variables			Independent variables					
			Time		Group		Time × group	
			F	*p*	F	*p*	F	*p*
Training load	sRPE based	ATL (au)	2.974	<0.001**	5.473	0.033*	0.909	0.568
		CTL (au)	2.018	0.018*	6.610	0.021*	1.627	0.075
		ACWR (au)	1.382	0.164	0.088	0.767	1.208	0.271
		skill-based session (au)	17.976	<0.001**	5.671	0.030*	0.891	0.591
		fitness-based session (au)	36.177	<0.001**	3.031	0.100	0.791	0.710
		skill-to-fitness ratio (au)	28.206	<0.001**	1.023	0.327	0.869	0.616
		Monotony (au)	12.412	<0.001**	11.182	0.004**	0.394	0.988
		Strain (au)	10.975	<0.001**	9.364	0.008**	0.574	0.916
	Duration based	ATL (au)	2.853	<0.001**	7.756	0.013*	0.781	0.723
		CTL (au)	2.910	<0.001**	6.934	0.018*	1.312	0.203
		ACWR (au)	1.632	0.073	0.158	0.691	1.235	0.252
		skill-based session (au)	18.729	<0.001**	8.849	0.009**	0.892	0.589
		fitness-based session (au)	47.207	<0.001**	3.171	0.093	0.600	0.899
		skill-to-fitness ratio (au)	36.998	<0.001**	0.824	0.365	1.250	0.222
		Monotony (au)	12.734	<0.001**	14.369	0.002**	0.380	0.990
		Strain (au)	10.780	<0.001**	15.299	0.001**	0.443	0.977
Physical fitness	Squat 1RM (kg)		2.745	0.055	0.788	0.388	0.798	0.502
	Bench press 1RM (kg)		9.893	<0.001**	0.243	0.629	0.261	0.853
	Run-up vertical jump (cm)		0.365	0.779	1.150	0.299	0.228	0.876
	5.8M × 6 rounds shuttle run (seconds)		12.270	<0.001**	2.083	0.168	1.179	0.329
	15M × 17 rounds shuttle run (seconds)		0.214	0.886	3.933	0.064	0.772	0.516

Abbreviations: sRPE, session rating of perceived exertion; ATL, acute training load; CTL, chronic training load; ACWR, acute chronic workload ratio; RM, repetition maximum.

Note: Bold indicates statistically significant values (*p* < 0.05).

*p < 0.05.

**p < 0.01.

### 3.3 Physical fitness

It was found that previous injury status had no significant main effect on the squat 1RM (*p* = 0.388), bench press 1RM (*p* = 0.629), 5.8 m × 6 rounds shuttle run (*p* = 0.168), and RVJ (*p* = 0.299). However, there was a slightly significant effect on the 15 m × 17 rounds shuttle run (*p* = 0.065). Further analysis revealed that the low-risk group performed significantly better than the high-risk group on the third (*p* = 0.034, MD = 1.41, 95% CI: 0.12–2.71) and fourth tests (*p* = 0.019, MD = 1.59, 95% CI: 0.30–2.88) of the 15 m × 17 rounds shuttle run. Additionally, no significant interaction was found between previous injury status and time on physical fitness indicators (*p* > 0.05).

### 3.4 Injuries

It was found that the incidence of injuries (*p* = 0.032, MD = 6.69, 95% CI: 0.68–12.70) and non-contact injuries (*p* = 0.034, MD = 6.11, 95% CI: 0.52–11.70) was significantly higher in the high-risk group (Table 3). There was no significant difference in the incidence of contact injury between the two groups (*p* > 0.05). Notably, players in the high-risk group were more prone to losing training time due to non-contact injuries (*p* = 0.026, MD = 4.46, 95% CI: 0.64–8.27).

**TABLE 3:** The descriptions and differences in the incidence of injuries and time loss between the high-risk and low-risk groups.

	High risk	Low risk	*p*	MD	95% CI of MD	Cohen’s d effect size
Injury Incidence (per 1,000 training hours)	11.70 ± 6.01	5.01 ± 5.63	0.032*	6.690	0.682 to 12.697	1.150
NCI Incidence (per 1,000 training hours)	9.38 ± 6.32	3.27 ± 4.80	0.034*	6.110	0.517 to 11.703	1.090
CI Incidence (per 1,000 training hours)	2.32 ± 2.40	1.74 ± 1.89	0.577	1.017	−1.587 to 2.746	0.269
LT-Injury (days)	9.09 ± 5.74	8.29 ± 15.05	0.896	0.805	−13.204 to 14.814	0.071
LT-NCIs (days)	4.96 ± 5.54	0.50 ± 1.32	0.026*	4.455	0.644 to 8.266	1.106
LT-CIs (days)	4.14 ± 5.33	7.07 ± 15.56	0.645	−2.935	−17.389 to 11.519	−0.252

Abbreviations: NCI, non-contact injury; CI, contact injury; LT-NCIs, loss of time due to non-contact injuries; LT-CIs, loss of time due to contact injuries; MD, mean difference.

Note: Bold indicates statistically significant values (*p* < 0.05).

*p < 0.05.

## 4 Discussion

This study retrospectively investigated the impact of previous injuries on subsequent training loads, fitness, and injury. Our findings revealed the following characteristics among players classified in the high-risk group compared to the low-risk group: (i) ATL, CTL, skill-based training load, training monotony, and training strain were significantly lower in the high-risk group. (ii) Players in the high-risk group took longer to complete the 15 m × 17 rounds shuttle run test, but this difference did not reach statistical significance. (iii) Incidence of injury, particularly non-contact injury, was significantly higher in the high-risk group. Meanwhile, players in the high-risk group experienced more training time lost due to non-contact injury.

The findings of this study provide evidence supporting that previous injuries not only increase players’ susceptibility to subsequent injuries but also adversely affect their training tolerance, which aligns with the basic hypothesis of this study (Mandorino et al., 2023). It is widely acknowledged that players who maintain high chronic workloads usually achieve the necessary fitness for competition, reducing their likelihood of injury (Windt and Gabbett, 2017). Conversely, players with low chronic workloads may lack the fitness reserves needed to handle the demands of intense competition, resulting in a higher risk of injury (Gabbett, 2016; Windt et al., 2017). However, some investigations yielded conflicting findings, leading to ongoing debate (Mohr et al., 2023; Impellizzeri et al., 2020; Suarez-Arrones et al., 2020; Myers et al., 2020). It is important to note that existing research has ignored the impact of previous injuries on training load and injury, which could be a crucial factor in explaining these inconsistencies (Sedeaud et al., 2020). Our findings revealed that players with a higher burden of previous injuries exhibited lower training loads (both acute and chronic workloads) and a higher incidence of subsequent injuries. Meanwhile, these players had lower training monotony and strain, which may mean that they could not withstand rapid changes in training load. To our knowledge, training monotony and strain are strongly associated with increased risk of injury, overtraining syndromes, and illness (Curtis et al., 2021). For instance, Brink and colleagues identified a significant correlation between higher monotony and an increased likelihood of injury (Brink et al., 2010). Nevertheless, our findings observed that players with a higher injury burden generally had lower levels of monotony and strain, but still had a high incidence of subsequent injury. These findings prompt the question: Does frequent exposure to higher chronic workloads reduce players’ risk of non-contact injuries? We propose that players with lower chronic workloads may have higher injury burdens than those with higher chronic workloads, making them more susceptible to maladaptation due to training intolerance and compensatory movement patterns (Opar et al., 2012; Croisier, 2004; Blyton et al., 2023; Hodges and Tucker, 2011; Desai and Gruber, 2021). As Howe and colleagues found, exercise-induced fatigue can exacerbate compensatory strategies, elevating the risk of injury to the affected limb (Howe et al., 2021). This vulnerability increases the susceptibility of these players to non-contact injuries, perpetuating a detrimental cycle. The debate will continue as existing research remains insufficient to provide a simple and definitive answer to this question.

Contrary to expectations, this study did not find an effect of previous injuries on the players’ strength, agility, or jumping ability. In theory, previous injuries should impair a player’s motor function, especially proprioception and neuromuscular control, leading to poorer fitness (Mandorino et al., 2023; Ritzmann et al., 2022). For example, Areia and associates found deficits in muscle activity, proprioception, and functional asymmetry in individuals with a history of hamstring strain during eccentric testing (Areia et al., 2019). Similarly, Bramah and colleagues observed differences in running kinematics among male runners with recurrent calf muscle strain injuries, possibly due to neuromuscular deficits (Bramah et al., 2021). However, contrary to earlier findings, no effect of previous injury on physical fitness was detected. One possible explanation is that players who have experienced previous injuries can maintain their performance through compensatory movements. For instance, youth basketball players with a history of injury exhibited similar peak mean vertical ground reaction forces during jump landings compared to uninjured players (Louw et al., 2006). However, players with a history of injury demonstrated smaller hip and knee flexion angles and greater eccentric activity during landing. Another possible explanation is that variations in test selection may have led to inconsistent results. A recent systematic review found that inter-limb strength asymmetry, measured by isometric knee extensor strength testing, correlates with higher injury risk (Guan et al., 2022). However, this correlation was not observed in unilateral jump test measures (Brumitt et al., 2020). Therefore, future observations will take into account the differences between the tests.

Notably, this study found that previous injuries slightly affected players’ repetitive sprint performance, similar to earlier findings (Røksund et al., 2017). Since repeated sprinting ability is important for basketball success and demands a high level of neuromuscular resistance to fatigue (Gottlieb et al., 2021; Charron et al., 2020), it was speculated that players with higher injury burdens would be more susceptible to neuromuscular fatigue. The research by Norte and colleagues supports our speculation (Norte et al., 2018). They found that patients who had experienced anterior cruciate ligament reconstruction (ACLR) exhibited impaired quadriceps strength and corticospinal excitability at each time point of knee extension. Frank and others found that neuromuscular fatigue may influence the increased risk of future injuries in women with a history of ACLR by altering lower extremity biomechanics and postural control (Frank et al., 2014). Despite lacking clinical information on neuromuscular fatigue, our study revealed significant differences between high-risk and low-risk groups in the third and fourth tests. Considering the effect of training intensity schedules on physical fitness, training-induced neuromuscular fatigue could be crucial in the relationship between previous injuries and a player’s ability to repeat sprints.

In summary, although previous injuries do not significantly affect the physical performance of young female basketball players, they can hinder training tolerance and increase the risk of further injuries. These findings suggest that coaches should adjust the training load composition based on previous injuries to improve players’ training tolerance and reduce the likelihood of additional injuries caused by neuromuscular fatigue, such as increasing the neuromuscular training, improving movement patterns and developing a fitness-fatigue model to manage the training load effectively (Paravlic et al., 2024; Šiupšinskas et al., 2019; Imbach et al., 2022). Nevertheless, given the information available, this study cannot offer more detailed recommendations for future training until the following limitations are addressed. Firstly, although the implemented sRPE-based training load quantification method is widely used in sports practice and research, it is a general indicator and does not differentiate between specific physiological response changes and intensity (Dhahbi et al., 2024). Secondly, this study was conducted within a sports management center, generalisations beyond this specific cohort should be treated with caution. While using data from a single centre can help avoid confounding factors such as youth maturity, coaching leadership styles, and match demands (whether in-season or off-season), it also limits the possibility that these findings can be extrapolated to all youth populations. Further research is needed to investigate the effects of the above-mentioned confounders on the findings (Silva et al., 2022; Giuriato et al., 2023). Finally, it would have been interesting to evaluate the effect of previous injury status on physiological responses following physical fitness testing, such as electromyography during strength testing and blood lactate during repetitive sprint performance testing (Kubo et al., 2019; Zebis et al., 2022).

## 5 Conclusion

The findings of this preliminary study revealed that while previous injuries do not substantially impact the physical fitness performance of young female basketball players during field-based assessments, they can impair subsequent training loads and lead to further injuries. These findings offer valuable insights into optimizing training loads based on previous injuries. Nevertheless, this study still has some flaws. Future research should include more comprehensive neuromuscular tests to investigate how previous injuries impact subsequent training loads and injuries, which could help develop injury prevention strategies based on training load management.

## Data Availability

The original contributions presented in the study are included in the article/Supplementary Material, further inquiries can be directed to the corresponding author.
