# Supplementary figures and images for: Investigating the effects of previous injury on subsequent training loads, physical fitness, and injuries in youth female basketball players
Source: Front Physiol. 2025 Jan 23;16:1506611. doi: 10.3389/fphys.2025.1506611 (PMC11798967; doi:10.3389/fphys.2025.1506611)

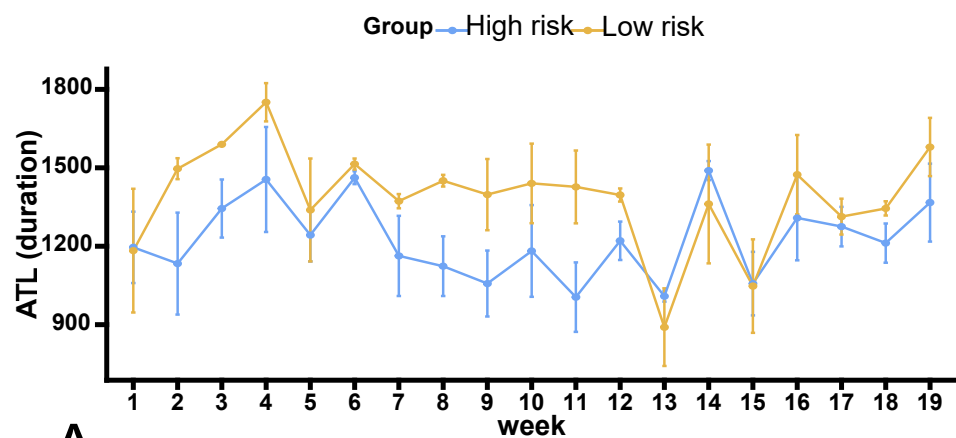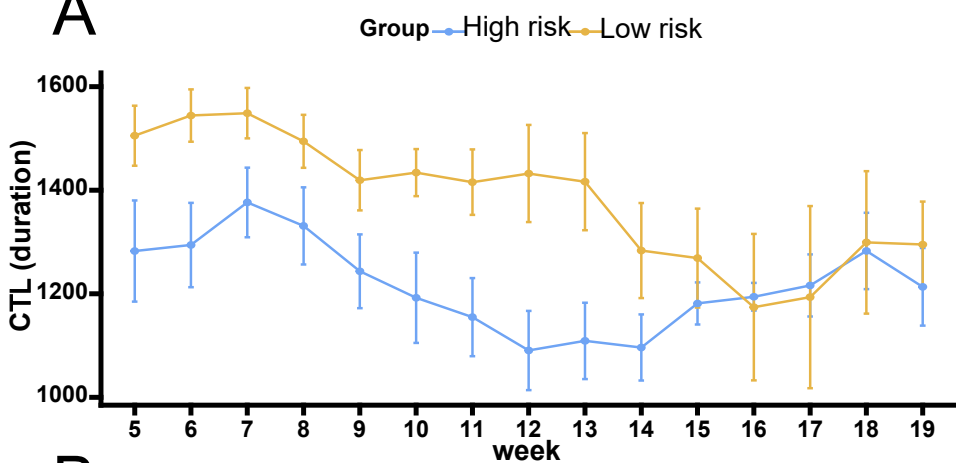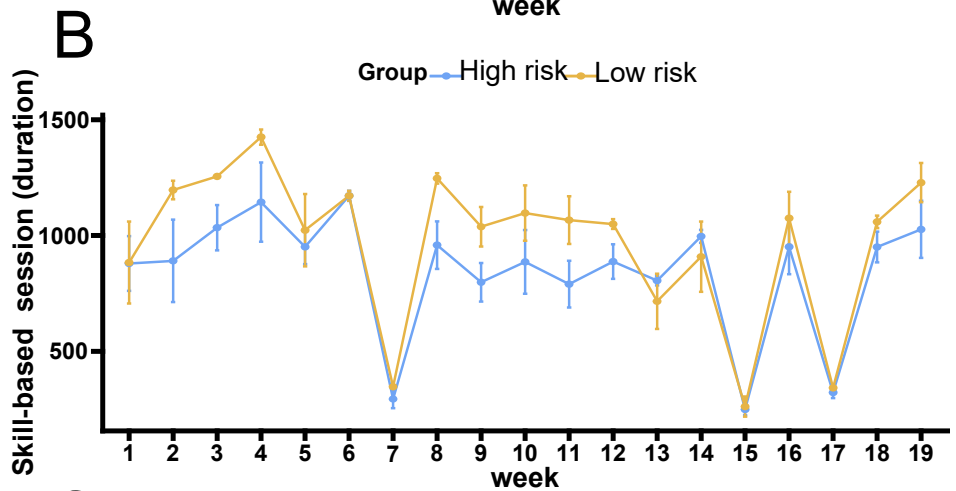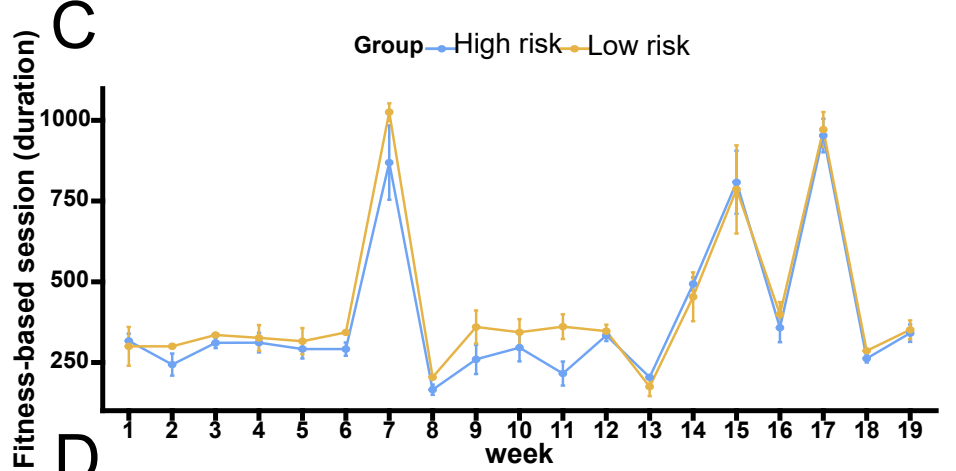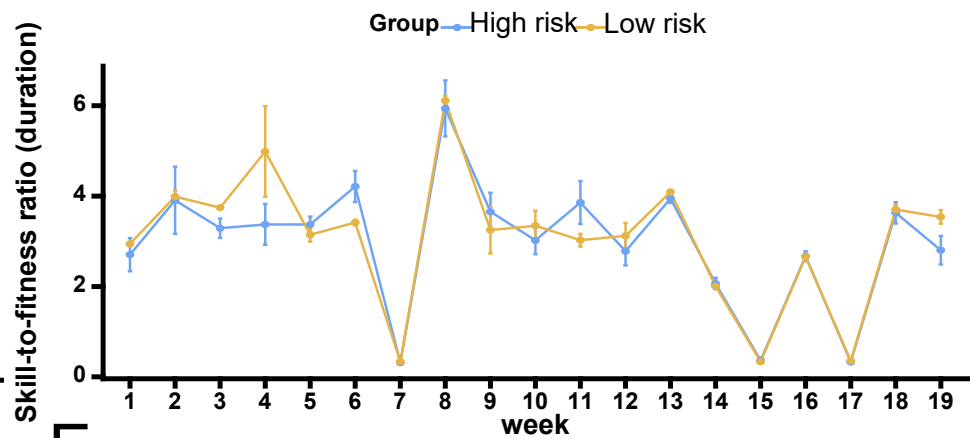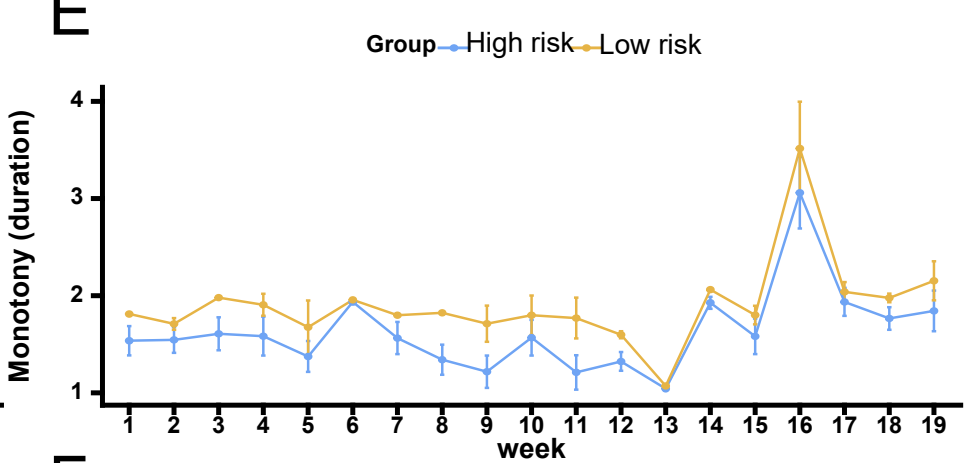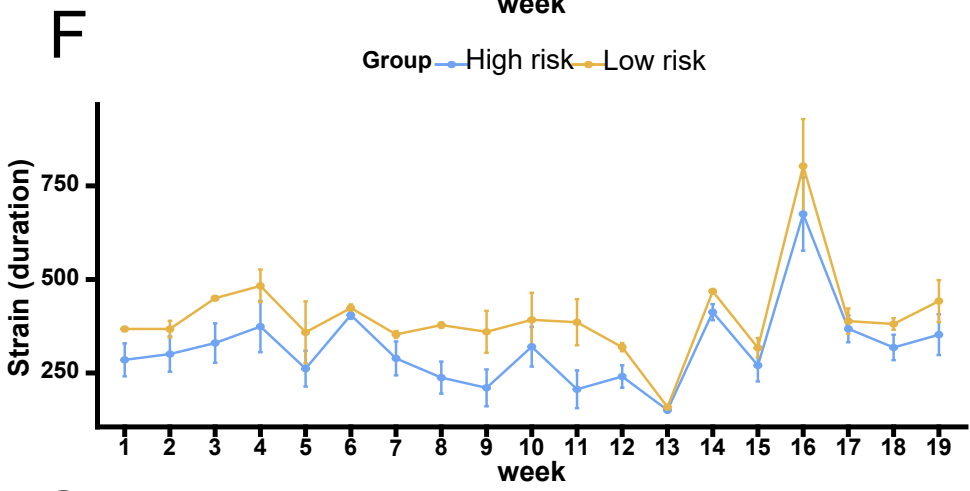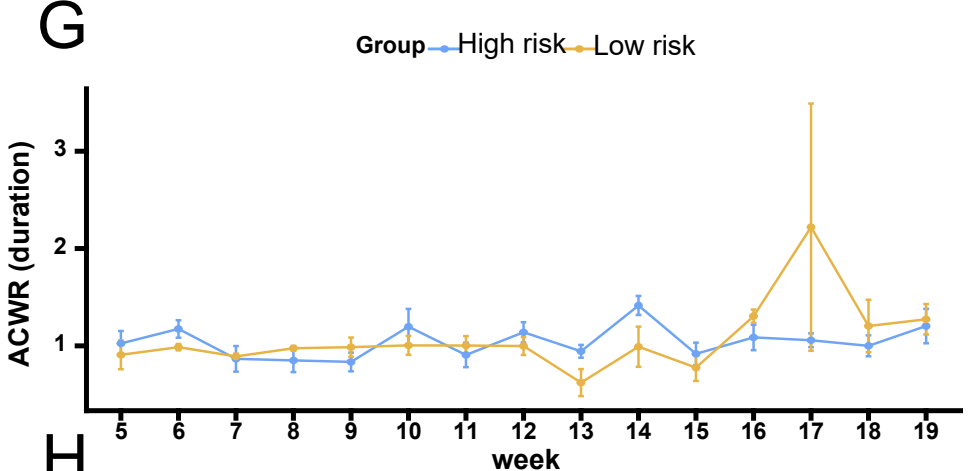

Supplement: Supplementary file 1 [file DataSheet2.pdf]

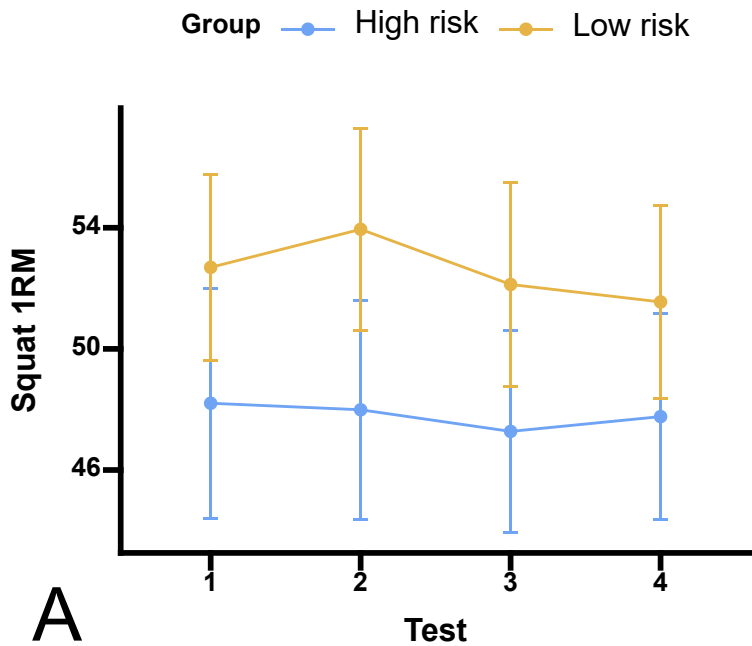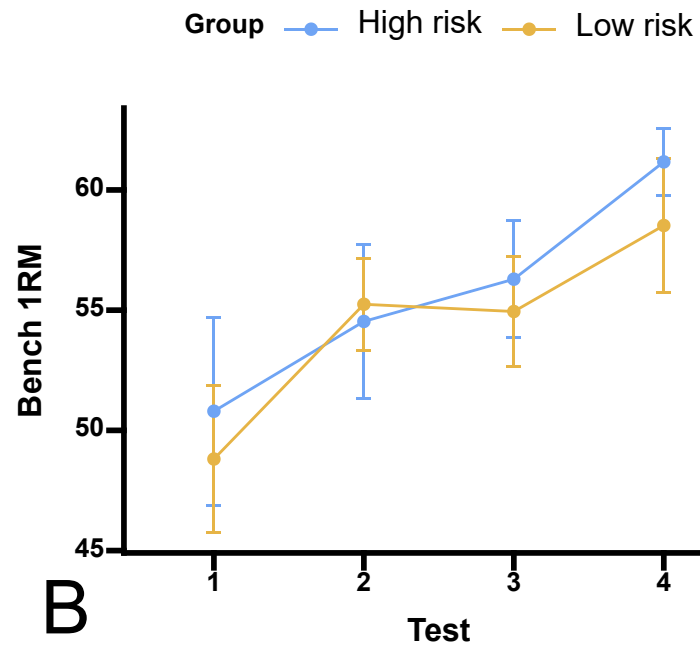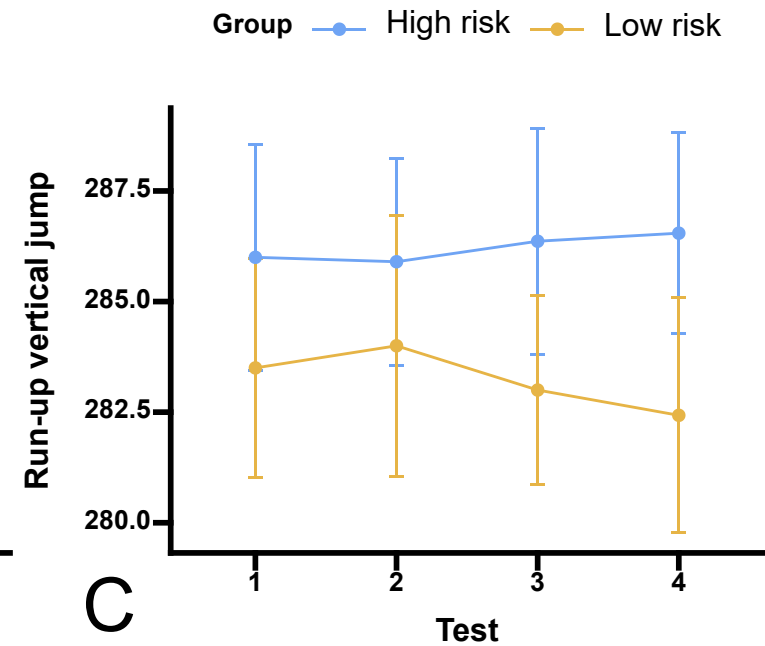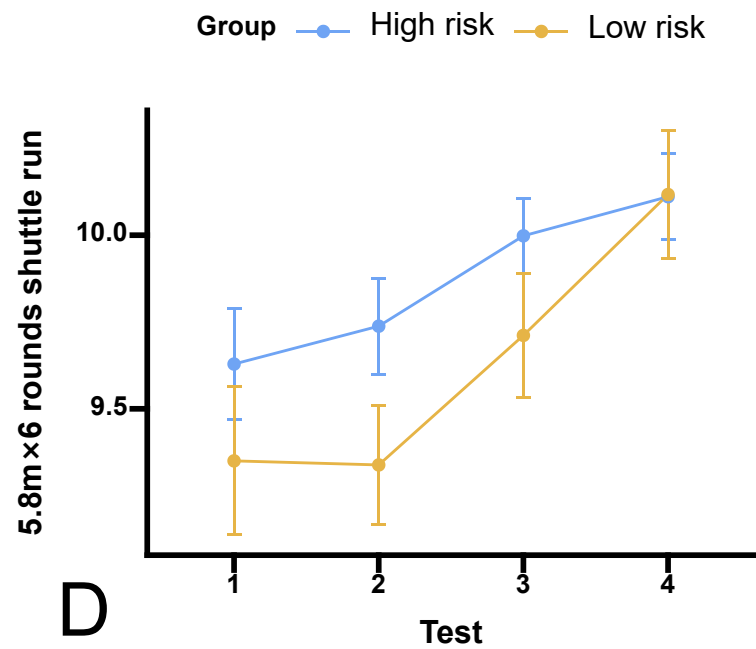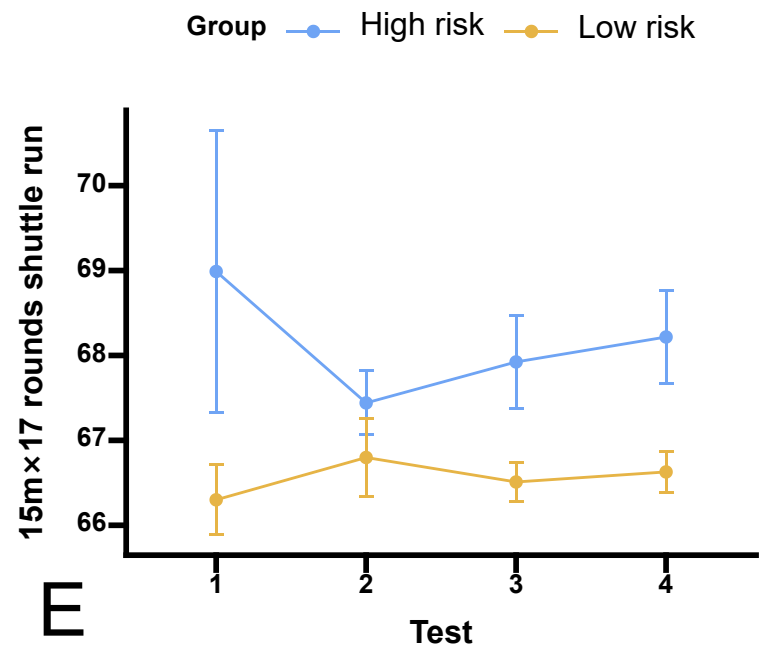

Supplement: Supplementary file 2 [file DataSheet3.pdf]

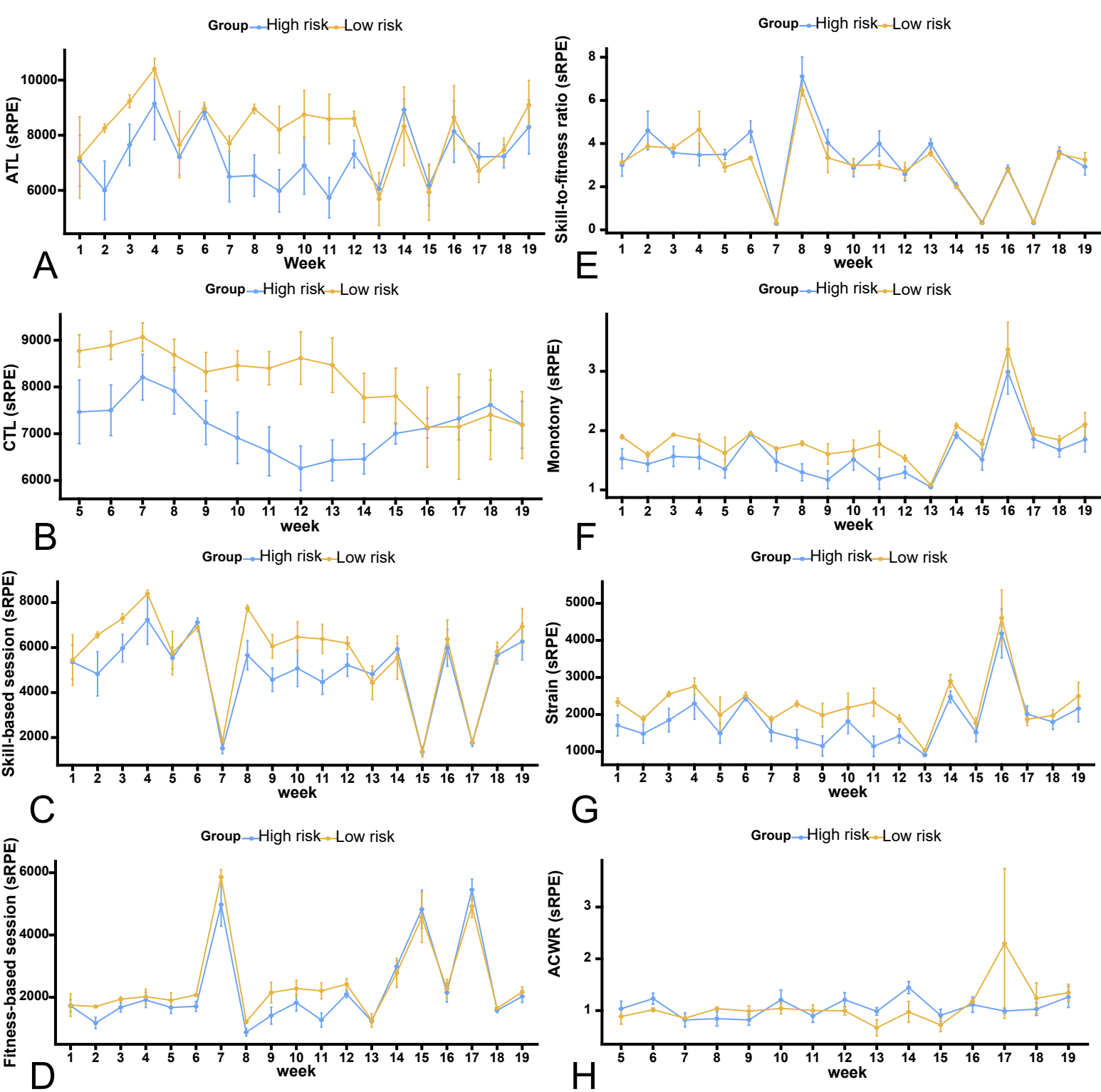

Supplement: Supplementary file 3 [file DataSheet1.pdf]
